# Development of EST-SSR markers and association mapping with floral traits in *Syringa oblata*

**DOI:** 10.1186/s12870-020-02652-5

**Published:** 2020-09-21

**Authors:** Yunyao Yang, Ruiqing He, Jian Zheng, Zenghui Hu, Jing Wu, Pingsheng Leng

**Affiliations:** 1 Beijing Advanced Innovation Center for Tree Breeding by Molecular Design, Beijing University of Agriculture, Beijing, 102206 China; 2 College of Landscape Architecture, Beijing University of Agriculture, Beijing, 102206 China; 3 Beijing Laboratory of Urban and Rural Ecological Environment, Beijing, 102206 China

**Keywords:** *Syringa oblata*, EST-SSR, Genetic diversity, Population structure, Association mapping

## Abstract

**Background:**

Lilac (*Syringa oblata*) is an important woody plant with high ornamental value. However, very limited genetic marker resources are currently available, and little is known about the genetic architecture of important ornamental traits for *S. oblata*, which is hindering its genetic studies. Therefore, it is of great significance to develop effective molecular markers and understand the genetic architecture of complex floral traits for the genetic research of *S. oblata*.

**Results:**

In this study, a total of 10,988 SSRs were obtained from 9864 unigene sequences with an average of one SSR per 8.13 kb, of which di-nucleotide repeats were the dominant type (32.86%, 3611). A set of 2042 primer pairs were validated, out of which 932 (45.7%) exhibited successful amplifications, and 248 (12.1%) were polymorphic in eight *S. oblata* individuals. In addition, 30 polymorphic EST-SSR markers were further used to assess the genetic diversity and the population structure of 192 cultivated *S. oblata* individuals. Two hundred thirty-four alleles were detected, and the PIC values ranged from 0.23 to 0.88 with an average of 0.51, indicating a high level of genetic diversity within this cultivated population. The analysis of population structure showed two major subgroups in the association population. Finally, 20 significant associations were identified involving 17 markers with nine floral traits using the mixed linear model. Moreover, marker SO104, SO695 and SO790 had significant relationship with more than one trait.

**Conclusion:**

The results showed newly developed markers were valuable resource and provided powerful tools for genetic breeding of lilac. Beyond that, our study could serve an efficient foundation for further facilitate genetic improvement of floral traits for lilac.

## Background

Lilac, which belongs to the genus *Syringa* (family Oleaceae), is an important woody ornamental plant. Approximately 27 wild species of *Syringa* have been described, most of which are native to China [1]. China is the world’s distribution center and center of origin for lilac with a cultivation history of 1000 years. After almost 100 years of breeding, there are approximately 2000 lilac cultivars, which show a wide range of forms and colors, and are commonly cultivated throughout the world [2]. *S. oblata,* a perennial deciduous shrub, is cultivated most widely as a garden plant in northern China due to its early flowering, elegant color, unique fragrance, and strong resistance to drought and cold [3]. As a native and early flowering tree species in the Beijing area, *S. oblata* was identified as an important species for courtyard greening [1].

Similar to other woody flowers, many new varieties of lilac have been bred using traditional breeding methods [4]. Although the traditional cross-breeding plays an important role in improving the ornamental traits of lilac, it can not adapt to the rapid development of modern lilac industry because of the long breeding cycle and the large amount of resources needed to cultivate new varieties [5]. Meanwhile, *S. oblata* is an outcrossing plant, many important ornamental traits are quantitative which are easily affected by the environment. In addition, little is known about the genetic structure of important ornamental traits of *S.oblata*, such as florescence, petal color and inflorescence etc. Therefore, it implies that there is an urgent need to understand the genetic structure of the important traits and shorten the lilac breeding cycle by modern tools to enhance breeding process.

Marker-assisted selection (MAS) breeding shorten the required time by directly selecting the targeted genotypes, thus accelerating the process and enhancing breeding efficiency [6]. An important basis for MAS is to determine the molecular markers associated with target traits. However, the genetic selection and breeding of lilac has been hindered to some extent by the lack of markers linked to target traits. Association mapping are an effective way understand the complex quantitative traits and the underlying genetic variation [7]. In recent years, it has been applied to the research of important quantitative trait loci mining in ornamental plants, such as *Helianthus annuus* [8], *Prunus mume* [9], and *Lagerstroemia indica* [10]. These studies showed that association mapping could effectively find the molecular markers closely linked to the genes controlling the target traits, and laid the foundation for the MAS. Moreover, association mapping relied on existing rich natural variation, resulting in high mapping accuracy [11].

With the development of molecular biology, many types of molecular markers have been developed and widely used for MAS breeding of ornamental plants [12–14]. Molecular markers such as allozymes and amplified fragment length polymorphism (AFLP) markers have been used to analyze the genetic diversity of *Syringa* and its related species [15, 16], which laid a theoretical foundation for *Syringa* breeding. However, compared with these DNA markers, simple sequence repeats (SSRs) markers are considered to be ideal molecular markers in MAS due to their advantages of co-dominant, multi-allelic, stability, extensive genome coverage, and ease of detection [17]. SSR markers, also known as microsatellite markers, are short tandem repeats of one to six nucleotides, which are widely distributed in coding and non-coding regions of the eukaryotic genome [18]. Over the past years, SSRs were successfully developed in different ornamental plants via data mining, such as *Magnolia ashei* [19], *Lagerstroemia indica* [20], *Paeonia suffruticosa* [21], and *Rosa hybrida* [22]. In addition, SSRs have also been widely used in plant genetics and breeding for genetic diversity analysis [23], cultivar identification [24], DNA fingerprinting [25], linkage mapping [26], and association mapping [27]. So far, only 14 pairs of polymorphic SSR markers have been developed for *S. vulgaris* [28] and *S. josikaea* [29], through conventional methods, however, it is greatly limited due to a prevalent lack of genomic and transcriptomic information. Currently, no SSR markers has been reported for *S. oblata.* Hence, it is still necessary to develop SSR markers for MAS in lilac breeding.

In recent years, next-generation sequencing (NGS) technology enabled the development of a large number of SSR, based on abundant transcript sequences [30–32]. Our laboratory has previously investigated the transcriptome of *S. oblata* flowers at different developmental stages by using RNA-seq technology and identified a total of 104,691 unigenes (accession: SRP063913) [33]. At the same time, a group of 192 unrelated individuals of cultivated *S. oblata*, exhibiting abundant phenotypic variations in floral traits, were sampled for association mapping. The objectives of this study were to (I) develop expressed sequence tag-SSR (EST-SSR) markers via *S. oblata* transcriptome sequences; (II) evaluate the genetic diversity and population structure of *S. oblata* cultivated populations; (III) to identify SSRs markers associated with floral traits, and (IV) to explore allelic effects on the natural variation of floral traits. Our results offer valuable resource for studies of population genetics in *S. oblata*, and will facilitate the speed of the screening the genotypes, which aims to provide a platform for MAS breeding.

## Results

### Frequency and distribution of EST-SSRs in *S. oblata*

In this study, 104,691 unigenes with a total length of 89.3 Mb were scanned by the Simple Sequence Repeat Identification Tool (SSRIT) [34], and 10,988 potential EST-SSRs were discovered from 9864 (9.4%) unigenes, with an average of one SSR per 8.13 kb. Among these, 977 unigenes contained more than one EST-SSR loci. Of these potential SSRs, di-nucleotide repeats were most abundant with a frequency of 32.86% (3611) followed by penta-nucleotide (23.25%, 2555), tri-nucleotide (18.08%, 1986), hexa-nucleotide (15.06%, 1655) and tetra-nucleotide repeats (10.75%, 1181) (Table 1).

**Table 1:** Summary of EST-SSR searching results in *S. oblata* transcripts

Searching items	Numbers
Total number of sequences examined	104,691
Total size of examined sequences (bp)	89,306,170
Total number of identified SSRs	10,988
Number of SSR containing sequences	9864 (9.4%)
Number of sequences containing more than 1 SSR	977
Frequency of SSRs	1/8.13 kb
Di-nucleotide	3611 (32.86%)
Tri-nucleotide	1986 (18.08%)
Tetra-nucleotide	1181 (10.75%)
Penta-nucleotide	2555 (23.25%)
Hexa-nucleotide	1655 (15.06%)

**Fig. 1:**
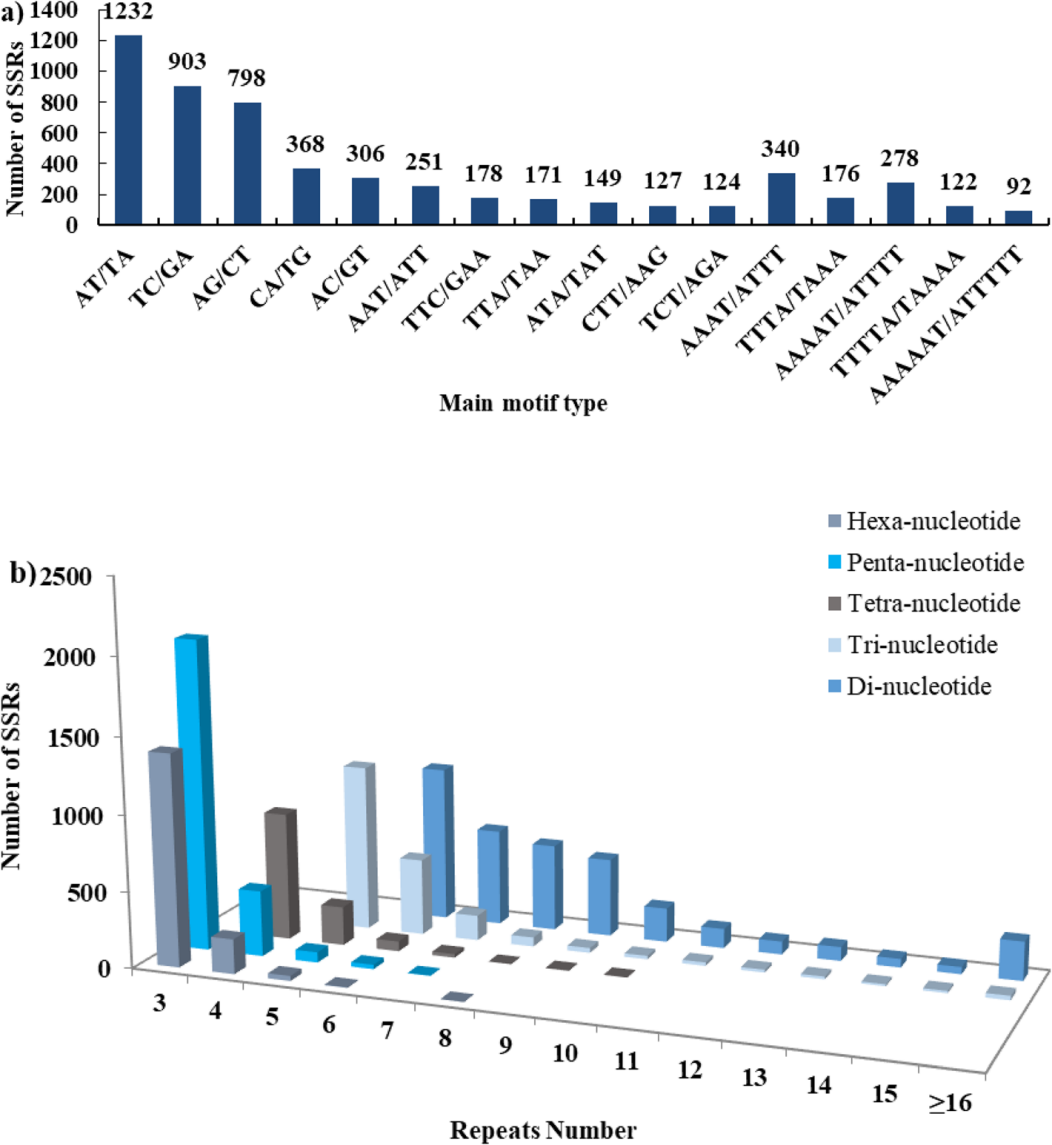
Characterisation of SSRs in *S. oblata* transcriptome. **a** number of main motif types. **b** number of different repeat motifs

The Fig. 1a showed the major types among repeat motifs. The most frequent type was AT/TA (11.21%, 1232) motif, followed by TC/GA (8.2%, 903), AG/CT (7.3%, 798), CA/TG (3.3%, 368), AAAT/ATTT (3.1%, 340), AC/GT (2.8%, 306), AAAAT/ATTTT (2.5%, 278), and AAT/ATT (2.9%, 251) (Fig. 1a). 96.6% of SSRs had length of 12 to 30 bp where 18 bp was the most common, while length of 3.4% of SSRs ranged from 31 to 75 bp. Figure 1b indicates that three tandem repeats (31.1%, 3418) were the most abundant followed by six (14.7%, 1610), four (1490, 13.6%), and five tandem repeats (13.1%, 1440). However, motifs of more than 15 tandem repeats were notably rare (2.16%) (Fig. 1b).

### EST-SSR markers development and polymorphic microsatellite loci screening

A total of 2042 EST-SSRs were selected for primer synthesis after the removal of ESTs with short flanking sequences or that were not suitable with primer design conditions. Among these, 932 (45.7%) primer pairs exhibited clear and repeatable bands, including 324 di-, 223 tri-, 88 tetra-, 161 penta-, and 136 hexa-nucleotides. Information about 932 EST-SSR primers is available in Table S1. In addition, 245 primer pairs produced fragments that were larger than expected. The remaining 865 primer pairs failed to produce any bands or produced multiple bands under different amplification conditions, which was likely due to assembly errors in sequences or primers. Thus, no further analysis was considered for them. All 932 primer pairs were selected for polymorphisms in eight *S. oblata* genotypes and 248 (12.1%) generated reproducible polymorphic products by PAGE, including 110 di-, 49 tri-, 18 tetra-, 47 penta-, and 24 hexa-nucleotides. The polymorphic ratio was 34.0, 22.0, 20.5, 29.2, and 17.6%, respectively.

### Genetic diversity and population structure

Thirty polymorphic EST-SSR markers in accordance with Hardy-Weinberg equilibrium were used to evaluate the genetic diversity and population structure of 192 *S. oblata* individuals. The results showed that 234 alleles were detected, and the mean number of alleles (*N*_*A*_) was 7.8, ranging from 3 to 16 (Table 2). Furthermore, the observed heterozygosity (*H*_*O*_) and expected heterozygosity (*H*_*E*_) ranged from 0.21 to 0.87 (mean 0.52) and 0.25 to 0.89 (mean 0.56), respectively. Among these, the *Ho* was lower than *H*_*E*_, indicating that inbreeding mainly affected the cultivated population of *S. oblata*. The Shannon’s information index for these loci ranged from 0.53 to 2.33 with an average of 1.15. The polymorphic index content (PIC) ranged from 0.23 for SO711 to 0.88 for SO525 with an average of 0.51. Nearly 93% (28) EST-SSR markers showed high or medium levels (PIC > 0.25) of genetic information, and only two markers (SO310 and SO711) had a low polymorphism level (PIC < 0.25). This suggests that these loci embodied a wealth of genetic information and that could be used for genetic diversity research on *Syringa* germplasms.

**Table 2:** Polymorphism information of 30 EST-SSR markers in 192 individuals. Number of alleles (*N*_*A*_); number of effective alleles (*N*_*E*_); Shannon’s information index (I); observed heterozygosity (*H*_*O*_); expected heterozygosity (*H*_*E*_); and polymorphism information content (PIC); untranslated region (UTR); coding sequence (CDS). All 30 polymorphic markers were at Hardy-Weinberg equilibrium (significance is *P* ≤ 0.01)

Primer name	SSR position	***N***_***A***_	***N***_***E***_	I	***H***_***O***_	***H***_***E***_	PIC
SO060	CDS	14	4.34	1.81	0.65	0.77	0.74
SO095	UTR	8	2.41	1.13	0.56	0.59	0.52
SO139	CDS	4	2.30	0.92	0.53	0.57	0.47
SO208	UTR	10	3.54	1.66	0.63	0.72	0.69
SO212	UTR	9	2.07	1.07	0.45	0.52	0.48
SO284	UTR	9	1.63	0.85	0.41	0.39	0.36
SO296	CDS	9	3.39	1.55	0.66	0.71	0.67
SO310	UTR	7	1.34	0.59	0.21	0.26	0.24
SO328	UTR	14	4.78	1.82	0.60	0.79	0.76
SO336	UTR	8	3.92	1.57	0.80	0.75	0.71
SO364	CDS	5	1.80	0.84	0.44	0.44	0.40
SO376	UTR	8	3.96	1.54	0.69	0.75	0.71
SO381	UTR	4	2.47	0.99	0.55	0.60	0.51
SO387	UTR	3	1.39	0.53	0.27	0.28	0.26
SO413	UTR	10	2.35	1.12	0.53	0.58	0.52
SO415	CDS	5	2.12	0.92	0.54	0.53	0.45
SO469	UTR	9	3.41	1.40	0.57	0.71	0.66
SO504	UTR	4	2.13	0.90	0.54	0.53	0.45
SO508	UTR	16	7.06	2.23	0.87	0.86	0.84
SO525	CDS	16	8.78	2.33	0.69	0.89	0.88
SO528	UTR	5	1.37	0.53	0.31	0.27	0.25
SO540	CDS	5	1.52	0.60	0.42	0.34	0.30
SO557	CDS	13	4.82	1.86	0.80	0.79	0.77
SO649	CDS	6	2.49	1.05	0.54	0.60	0.52
SO696	UTR	3	1.52	0.54	0.30	0.34	0.29
SO711	CDS	6	1.33	0.53	0.28	0.25	0.23
SO783	UTR	10	3.17	1.4	0.57	0.69	0.63
SO813	CDS	5	1.86	0.91	0.40	0.46	0.42
SO833	CDS	6	1.41	0.61	0.26	0.29	0.27
SO889	UTR	3	1.94	0.69	0.45	0.48	0.37
Mean		7.8	2.89	1.15	0.52	0.56	0.51

**Fig. 2:**
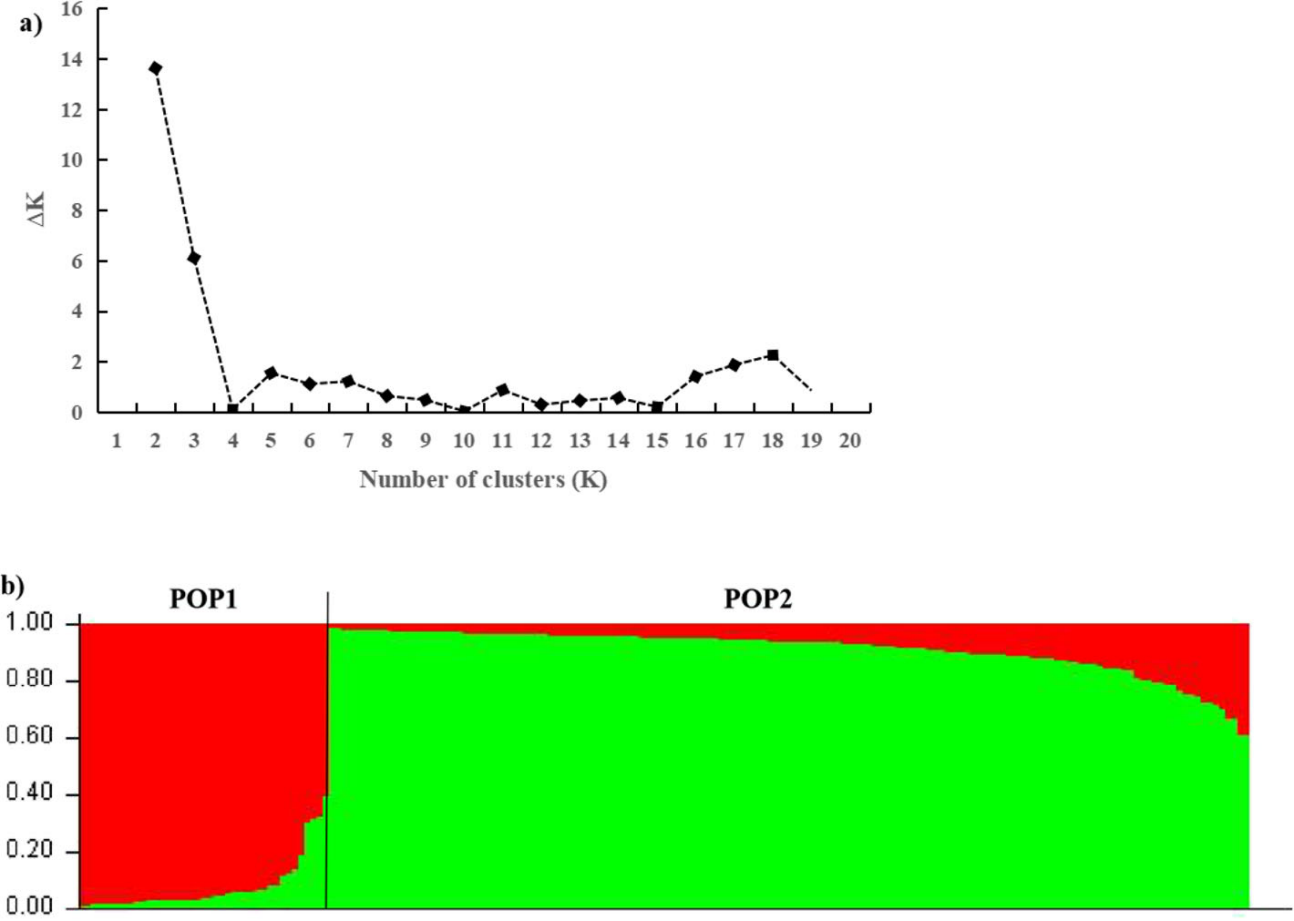
Population genetic structure of cultivated population of *S. oblata* (*K* = 2). **a** The relationship between the number of clusters (*K*) and the corresponding *ΔK* statistics based on STRUCTURE analysis. **b** The vertical coordinate of each group indicates the membership coefficients for each genotype. Different colors represent genetic stock

The existence of population structure will lead to the increase of linkage disequilibrium (LD) level, which may result in the correlation between the target traits and unrelated loci. Thus, the analysis and adjustment of population structure is the premise of carrying out association analysis. The population structure of 192 individuals was analyzed based on 30 polymorphic markers via STRUCTURE 2.3.4 and a clear peak was obtained at the value *K* = 2 (Fig. 2a) using the statistical model of Evanno et al. [35]. Accordingly, the 192 individuals can be divided into two subpopulations, that is, POP1 specified in red (41 individuals) and POP2 specified in green (151 individuals). As shown in Fig. 2b, each individual is represented by a thin vertical line and classified according to its estimated membership probability (*Q*), which was used for the structure-based association mapping. As an alternative strategy to using the STRUCTURE algorithm, principal component analysis (PCA) is widely used to identify population subpopulations. The PCA separated the association population into two subpopulations (Figure S1), which the clustering results were similar to the clustering results obtained using STRUCTURE.

### Phenotypic traits analysis and single-marker associations

The variance degree of the target traits in population was an important parameter for association mapping. The values of variation coefficient of quantitative traits ranged from 19.72 to 30.22% and the statistical values of the distributions were presented in Table S2. Moreover, correlation analysis between different traits showed 14 significant correlations (*P* < 0.05), of which 12 showed a highly significant correlation (*P* < 0.01) (Table S3). Of these, inflorescence length, inflorescence width, corolla lobe length, corolla lobe width and corolla tube length all had highly significant positive correlations. In addition, the highly significant positive correlations were observed between corolla lobe (state) and corolla lobe (periphery). More details of the phenotypic correlations among the nine traits in the association population were presented in Table S3. Meanwhile, Q cluster analysis for nine phenotypic traits of 192 individuals showed that the population divided into two subgroups, which was similar to the results of STRUCTURE basing EST-SSR markers (Figure S2).

**Fig. 3:**
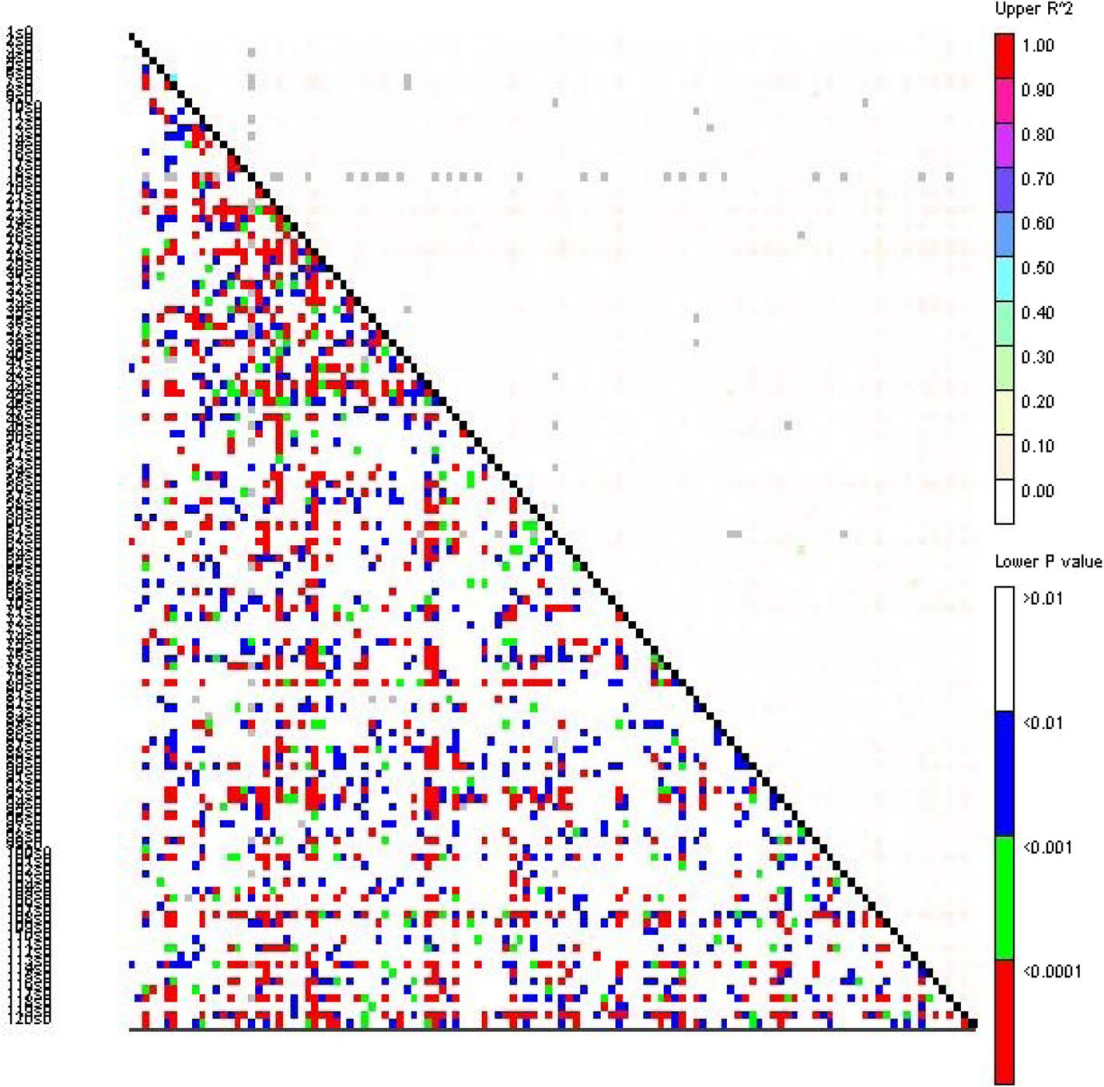
Pairwise linkage disequilibrium (LD) (*r*^2^) between SSR markers. Most of the markers were in a significant linkage equilibrium (*r*^2^ < 0.1; *P* < 0.001), and several loci were in significant LD, such as markers SO015-SO428 (*r*^2^ > 0.3; *P* < 0.001)

For association mapping, understanding the patterns of LD is an important prerequisite. One hundred nineteen polymorphic markers with minor allele frequency (MAF) > 1% were used to analyze the LD level in the 192 cultivated *S. oblata* individuals. The results showed that the *r*^2^ ranged from 0.0001 to 0.5154 for all loci pairs. The LD level was low and most of the markers were in linkage equilibrium (*r*^2^ < 0.1; *P* < 0.001). Eight hundred ninety-nine loci pairs had linkage disequilibrium (*P* < 0.001), and 830 had *r*^2^ > 0.005 (83.1%) (Fig. 3). Of course, it was also found that there was a strong LD level among some SSR loci, such as markers SO015-SO428 (*r*^2^ > 0.3; *P* < 0.001).

The association analysis between 119 SSRs and nine traits was carried out based on the mixed linear model (MLM) model. A total of 1071 single-marker association tests were performed, of which 20 associations were significant (*P* < 0.01) following multiple test corrections using the false discovery rate (FDR) method at a significance level of *Q* < 0.01, involving nine traits with 17 SSRs. For each trait, the number of significant associations varied ranging from zero to four. These loci explained a phenotypic variance ranging from 0.36 to 20.76%, with an average rate of 5.69% (Table 3). Of these, four SSR markers were detected which were significantly associated with corolla lobe (state) and corolla lobe width, respectively. Corolla lobe (periphery) had three significant associations; inflorescence length, corolla lobe length and petal color had two significant associations each; one significant association each with inflorescence width, corolla tube and florescence were observed in the association population (*Q* < 0.01; Table 3). In the present study, three SSRs (marker SO104, SO695 and SO790) exhibited significant associations with multiple traits, suggesting the pleiotropic effect or the continuity of the genomic regions for certain traits. For eight of the 20 associations, the mode of gene action is consistent with under- or over dominance; the remaining 12 markers were separated between modes of gene action that were additive (7) or partially to fully dominant (6) (Table 3).

**Table 3:** Summary of significant SSR marker-trait pairs from the association test results in the *S. oblata* populations after correction for multiple testing errors. *P* value: significance level for association (significance is *P* < 0.01); *Q* value: a correction for multiple testing (FDR (*Q*) < 0.01); *R*^2^: percentage of the phenotypic variance explained; *a*: additive; *d*: dominance; *S*_*p*_*:* standard deviation for the phenotypic trait under consideration. The algorithm and formulas for gene action were calculated as previously reported [36, 37]

Trait	Locus	***P*** value	***Q*** value	***R***^**2**^ (%)	2***a***	***d***	***d***/***a***	2***a***/***S***_***p***_
**Inflorescence length**	SO208	2.61E-05	6.53E-05	3.42	1.264	5.016	7.934	0.046
	SO112	4.60E-08	2.48E-30	7.69	− 5.468	0.889	− 0.325	−0.200
**Inflorescence width**	SO627	6.21E-04	5.10E-29	7.29	−0.810	0.125	−0.308	−0.052
**Corolla lobe length**	SO608	3.60E-16	5.41E-26	4.30	−0.171	0.231	−2.696	−0.110
	SO695	8.95E-05	1.80E-15	4.59	−0.367	− 0.222	1.211	−0.235
**Corolla lobe width**	SO060	1.24E-31	1.15E-09	5.36	−0.705	0.116	− 0.328	− 0.705
	SO311	5.18E-04	1.53E-06	3.65	0.052	0.055	2.104	0.052
	SO531	3.45E-04	1.53E-06	0.36	4.043	0.362	0.179	4.043
	SO695	6.50E-03	7.16E-05	3.68	−0.161	−0.049	0.609	−0.161
**Corolla tube length**	SO649	3.58E-05	9.40E-05	20.76	−0.059	0.008	−0.258	−0.025
**Corolla lobe (state)**	SO790	5.35E-07	1.49E-04	0.93	1.602	0.913	1.140	2.347
	SO503	4.78E-04	7.16E-05	11.78	0.277	0.146	1.057	0.406
	SO415	5.17E-05	7.35E-04	2.30	0.182	0.208	2.288	0.267
	SO663	2.88E-10	7.40E-04	1.89	0.034	0.045	2.634	0.050
**Corolla lobe (periphery)**	SO104	1.60E-03	8.28E-04	4.61	0.794	0.196	0.493	0.989
	SO505	8.11E-27	8.70E-04	9.58	0.085	0.706	16.610	0.106
	SO331	6.96E-04	8.87E-04	7.08	0.290	0.022	0.150	0.361
**Florescence**	SO790	5.10E-30	1.78E-03	7.92	2.935	1.389	0.947	7.919
**Petal color**	SO104	7.54E-04	4.48E-03	2.62	0.074	−0.296	−8.008	0.056
	SO805	4.60E-03	6.50E-03	4.00	0.066	0.189	5.756	0.050

## Discussion

Deep transcriptome sequencing, which generates quantities of data exponentially, provides comprehensive information and good resources for developing new SSR markers and identifying novel genes [38–40]. Currently, a plenty of SSR markers, based on transcriptome sequences, have been successfully developed and used for functional variation detection and gene-related genetic analysis in many organisms, accelerating progress in MAS breedings [41–43]. In this study, we first reported the SSR markers of *S. oblata* basing transcriptome sequences generated via NGS technologies.

A total of 10,988 EST-SSR loci was identified from 9864 transcriptome sequences, representing approximately 9.4% of the transcriptome in *S. oblata*, which was consistent with previous reports that the loci frequency ranged from 2.65 to 16.82% in dicotyledons [44]. Moreover, the distribution density was one SSR per 8.13 kb, which was lower than previous reports for *Elymus sibiricus* (1/6.2 kb) [45] and *Mucuna pruriens* (1/5.3 kb) [46], but higher than *Medicago sativa* (1/12.06 kb) [47] and *Nelumbo nucifera* (1/13.04 kb) [48]. The frequency of SSR loci varied greatly among different species, probably related to the size of the data set, the SSR search criteria, and the utilized mining tools for SSR search. Previous findings have shown that di-nucleotide repeats are the most common type of SSR repeat in dicotyledons [44, 49–51], as well as this study, may due to over-expression of untranslated regions (UTRs) [49]. Among the di-nucleotides observed, AT/TA (34.1%) was the most abundant, similar to *Brassica* [52], which may be due to the high frequency of the amino acids Ile (AUA) and Tyr (UAU), because their codons carried these motifs [53]. Furthermore, in agreement with the low coverage of GC repeats in dicotyledons, the lowest frequency (0.2%) of GC/CG was found in *S. oblata*. It might be due to the methylation of cytosine, which inhibited transcription in a number of plants [54]. Through comparison, it was found that different species had different types of repetition and motif may due to different selection criteria.

Of the 2042 designed EST-SSR primers, 932 (45.7%) EST-SSR markers were successfully amplified. The amplification rate was higher than that of *Brassica campestris* (16.68%) [55], but lower than that of *Iris* (76%) [56] and *Chrysanthemum indicum* (88.6%) [57]. The amplification rate is slightly lower in this study, which may be related to intron content and strict primer selection criteria. As far as we know, hardly any research has been performed on the development of extensive EST-SSR markers in *Syringa*. Furthermore, 12.1% (248) EST-SSR markers were polymorphic among the eight *S. oblata,* which was lower than the polymorphic ratio of 76.6% in *Catharanthus roseus* [58] and 52.7% in *Melilotus albus* [59], respectively. The polymorphic ratio of EST-SSRs was at a relatively low level in this study, may be affected by the number of materials used and the narrow geographical origin.

To establish the foundation for association mapping and MAS breeding programs, we selected 30 polymorphic markers in accordance with HWE equilibrium to assess genetic diversity and population structure in *S. oblata*. A total of 234 alleles were obtained across 192 individuals with a mean number of 7.8 alleles per locus, which was higher than the level of *N*_*A*_ (3.44) within 75 genotypes of *S. vulgaris* that were determined using nine polymorphic SSR markers [28]. The mean number of alleles (NA) of 7.8 might be associated with the relatively large sample capacity and polymorphic SSR markers used. However, compared with *S. josikaea* (14.2), 7.8 alleles per locus is lower [29]. That the *H*_*E*_ was lower than *H*_*O*_ at nine SSR loci indicated a significant excess of heterozygotes at these loci. But *H*_*O*_ was lower than *H*_*E*_ at the other 20 SSR loci, indicating inbreeding in the population. PIC value is also an important measurement of genetic diversity, the SSR marker polymorphism between 0.23 to 0.88, with a mean of 0.51, in line with previous research results [60]. The analysis of the position of the 30 polymorphic SSR markers within the transcriptome sequences showed that 18 markers (60%) were present in the UTR. The degree of polymorphism of SSR loci used in this study is quite considerable, which may be due to the occurrence of most of the polymorphic SSRs in the UTR. Meanwhile, 15 out of 30 primers with the PIC greater than 0.5 indicated good informativeness as a marker. It indicated that the newly developed polymorphic EST-SSR markers were informative and effective for the further genetic analysis.

Estimating the population structure could avoid the false-positive associations to improve the efficiency of association mapping [61, 62]. Thus, the selection of subpopulations is critical to the results of association. For example, it has been reported that the selection of rice subpopulation structure determines whether the marker is strongly associated with the trait [63]. The STRUCTURE model explained the existence of HWE or LD by introducing the population structure and trying to find subgroups in equilibrium [64]. Thus, the population subsets in the structure analysis conformed to HWE, and two distinct subpopulations were obtained within 192 individuals. The pattern of individuals assigned to subgroups was consistent with its provenance. In the future research, more various wild genotypes will be used to design a LD mapping scheme, basing on the genetic diversity, to improve the utilization of the existing genetic resources of *S. oblata*. In addition, detailed knowledge of LD level of species in association populations is an important prerequisite in increasing the resolution of marker-trait associations. In this study, a low level of LD may be due to the fact that *S. oblata* is an outcrossing plant and its high recombination rate. Previous studies have shown that the LD of woody plants was very low [65, 66]. For instance, LD was found to rapidly decrease in the range of several kilobases in loblolly pine [67], and similar findings of limited LD among candidate genes were existed in other conifer species [68–71].

*S. oblata* is popular because of its early flowering, elegant color and distinctive scent. Abundant germplasm resources are the material basis of modern breeding, and the diversity of germplasm resources is directly related to their effective utilization. The analysis of phenotypic traits is the most basic approach to touch germplasm resources [72]. The coefficient of variation (CV) of five quantitative traits measured in this study ranged from 19.72 to 30.22%, which was consistent with previous reports on *S. oblata* [73, 74]. It is a long-term task for breeding workers to improve the floral traits of lilac and cultivate new varieties of lilac. Association mapping has been extensively used to determine associations between potential targeted loci and important traits [75–77]. Single-marker associations may be more powerful in this low LD tree species, comparing with the haplotype-based associations effecting from an individual significant marker [66]. In this study, an MLM model was employed to confirm the associated makers for floral traits basing on the single-marker associations, with the population structure and kinship as the covariance. Meanwhile, in order to further enhance the accuracy of the association results, FDR method was used to correct the *P* values for all associations on many occasions, which greatly reduce the inflation of *P* values. Finally, a total of 17 SSR markers were identified, which were significantly associated with floral traits in the association populations. We identified the multiple significant markers may be due to the markers were developed from the transcriptome sequence of *S. oblata* flower [33]. For many plants especially containing complex quantitative traits in association analysis, a huge challenge is that a generous number of loci have little influence [78]. Many significant associations were identified which partly interpretated a small part of the phenotypic variance, suggesting that many loci for genetic control are associated with relatively small individual effects. Similar results were reported in genetic studies of quantitative characters of woody plants, reflecting a polygenic quantitative model [79–82].

Interestingly, we found that markers SO104, SO695, and SO790 are significantly correlated with more than one trait, which is consistent with the significant phenotypic correlation between these traits. These pleiotropic associations may help to identify important genomic regions [7]. Knowing the genetic control of these flora traits makes it possible to further infer the importance of SSR related to characters [83]. Corolla tube length was associated with SO649 of additive model, explaining 20.76% of the phenotypic variance, which made this marker attractive in MAS breeding. Florescence and petal color were the important ornamental trait, which largely determines its ornamental value. Marker SO790 was significantly associated with florescence and revealed a pattern of gene action consistent with over-dominance. Petal color was highly significantly associated with marker SO104 and SO805, which showed a pattern of gene action an additive model, explaining the cumulative amount of phenotypic variance of 6.2%. The additive models which accumulated multiple SSR genetic effects were used to explain the obvious heritability of traits made MAS attractive in plant breeding [11]. Eight out of the 20 associations, the modes of gene action were in accordance with under−/over dominance. Compared with the corresponding homozygous individuals, heterozygous individuals contribute more to the ornamental value of the flowers in the effect of over-dominance allelic. Such individuals are conducive to preserve the genetic variability, in the context of population genetics, and may own a natural advantage due to containing both alleles (over-dominant selection) [84]. Meanwhile, to explore the potential function of the 17 polymorphic SSR-containing sequences, a search was executed in GenBank using BLASTX against 17 transcriptome sequences (Table S4). Ten sequences were matched to the *Olea europaea* var. *sylvestris* protein. The transcriptome sequence of the SO649 marker was annotated as E3 ubiquitin-protein ligase. The transcriptome sequence of the SO649 marker was a B3 domain-containing protein, which play extremely important role in stress responses and plant growth and development [85]. The transcriptome sequence of SO415 was annotated as transcription repressor *OFP15*, which was related to flower development in *Arabidopsis thaliana* [86]. In conclusion, some EST-SSR markers associated with floral traits were identified of *S. oblata* using association mapping approach, laying a basis for further analysis MAS breeding programs with the aim of ameliorating the floral traits.

## Conclusion

In summary, a total of 932 SSR markers were developed firstly in *S. oblata* including 248 polymorphic primers. These newly developed EST-SSR markers provided an important resource for genetic diversity, comparative genomics, gene-based association studies, and marker-assisted selection in *Syringa.* This is the first report about association mapping of *S. oblata* and a total of 17 SSR markers associated with floral traits were identified. These results will play an important role in future MAS breeding programmes.

## Methods

### Plant materials and DNA extraction

A collection of 1900 cultivated *S. oblata* individuals was obtained from Hebei and Liaoning in North China and cultivated in the field of Beijing anjiming Labor Co., Ltd. (40°15′N, 116°60′E), the scientific research of Beijing University of Agriculture, adopting standard agronomic cultivation measures. All plants were about 9 years old, grew well, and flowered normally, showing a stable combination of flower traits. Eight *S. oblata* individuals were randomly selected for SSR marker development and polymorphism marker screening. A set of 192 *S. oblata* individuals was selected from the collection as association population, exhibiting various flower colors and shapes. Genomic DNA was extracted from the young leaves using the DNA secure plant kit (Tiangen Biotech, Beijing, China). 2% agarose gels and NanoDrop ND-1000 UV/Visible spectrophotometer (Wilmington, DE) were used to test the DNA quality and quantity, respectively. The working concentration of DNA was diluted to 25 ng/μL.

### Phenotypic measurements

A total of nine traits including five quantitative traits and four qualitative traits were scored in 192 individuals of the association population, with at least five repeats each genotype. The floral traits including inflorescence length, inflorescence width, corolla lobe length, corolla lobe width, corolla tube length, corolla lobe (state), corolla lobe (periphery), florescence, and petal color, were measured at full bloom. The five quantitative traits were all gauged with digital caliper (YB5001B, Kraftwelle Industrial Co. Ltd., China). The petal color values were measured at five inflorescences (including 25 florets) each genotype by using the colorimeter (CR-400, Konica Minolta Holdings, Inc., Japan), and were divided into six groups (Table S5) according to the L*, a*, and b* measurements generating. Corolla lobe (state), corolla lobe (periphery) and florescence were measured according to the Guidelines for the Conduct of Tests for Distinctness, Uniformity and Stability - *Syringa.* All measurements were described in Table S5. The software SPSS statistics 20 was used for analysis of variance (ANOVA), phenotypic correlations and Q cluster analysis of the traits.

### Identification and validation of EST-SSRs

A total of 104,691 unigenes were obtained from *S. oblata* transcriptome sequences of three distinct stages of flower development. The RNA-seq data have been submitted to the NCBI Sequence Read Archive (SRP063913, https://trace.ncbi.nlm.nih.gov/Traces/sra/?study=SRP063913) [33]. The simple sequence repeat identification tool program (SSRIT) (http://www.gramene.org/db/markers/ssrtool) was used to search for SSRs within transcriptome sequences [34]. SSR search criteria was conducted based on perfect di-, tri-, tetra-, penta-, and hexa-nucleotide motifs minimum number of six, five, four, three, and three repeats, respectively. Primer Premier 5.0 software (Premier Biosoft International, Palo Alto, CA, USA) was used to design primers in the flanking regions of the SSRs. Primers were synthesized by Beijing Ruibio BioTech (Beijing, China). To predict whether the SSR was present in the coding sequence (CDS) or untranslated region (UTR), the ORF Finder software (http://www.ncbi.nlm.nih.gov/gorf/gorf.html) was used to identify the initiation and termination codons in the EST sequences.

Polymerase chain reactions (PCR) amplifications were conducted in a 10 μL reaction system containing 1 μL of 25 ng/μL DNA, 0.5 μL of 10 μM of each primer, 5 μL of 2 × Power Taq PCR Master Mix (Aidlab, Beijing, China), and 3 μL sterile distilled water. The PCR was performed under the following conditions: 95 °C for 5 min followed by 30 cycles of 95 °C for 30 s, the appropriate annealing temperature (Table S1) for 30 s and 72 °C for 1 min, and a final extension at 72 °C for 10 min. And then, the PCR products were tested on 2% agarose gels. Successful amplified products were separated on 6% denaturing polyacrylamide gel electrophoresis (PAGE) and then visualised using silver staining. To identify the repeatability of the results, each primer pair was amplified three times. Finally, a subset of polymorphic SSR primers was identified and specified as ‘validated markers’.

One hundred nineteen polymorphic SSR primers developed in this study were randomly selected to add fluorescent dye for population genotyping. All fluorescence primers were synthesized by Beijing Ruibio BioTech (Beijing, China). The products were separated by capillary electrophoresis using an ABI3730xl DNA Analyzer (Applied Biosystems, Carlsbad, CA, USA). The polymorphic loci analysis was carried out by the software GeneMapper version 2.2.0 with the LIZ 600 size standard (Applied Biosystems).

### Genetic diversity and population structure analysis

The genetic diversity parameters, including the number of alleles (*N*_*A*_), number of effective alleles (*N*_*E*_), the observed and expected heterozygosities (*Ho* and *H*_*E*_, respectively), and the Shannon information index for each marker, were calculated with POPGENE version 1.31 [87]. The polymorphism information content (PIC), Hardy-Weinberg equilibrium (HWE) and minor allele frequency (MAF) for each marker were calculated using PowerMarker version 3.25 [88].

The genetic structure of the 192 *S. oblata* individuals was assessed using STRUCTURE v2.3.4 basing the Markov chain Monte Carlo (MCMC) algorithm and the Bayesian framework. Assuming an admixture model sample, 20 independent runs were performed for each value of *K* ranging from 1 to 20, each with a length of burn-in period of 100,000, followed by 500,000 iterations. In this way, the population membership estimates (*Q* matrix) were extracted, supplying the membership percentage for association mapping. The PCA was assessed using the Multi-Variate Statistical Package (MVSP) version 3.1 (Kovach Computing Services, Anglesey, Wales, UK).

### Marker-trait associations

The linkage disequilibrium (LD) analysis was performed with TASSEL version 2.1 software using 10^5^ permutations to calculate the *r*^2^ value between pairs of SSR markers (minor allele frequencies > 1%) [89]. Pairs of loci were considered to have a significant LD when *P* was < 0.001. The mixed linear model (MLM) considering both the *Q* matrix and kinship matrix in TASSEL version 2.0.1 was used to reveal the association between floral traits and SSR markers. The *Q* matrix was obtained from STRUCTURE. The kinship matrix (*K*) was estimated using SPAGeDi software version 1.2 [90]. The significant threshold for selecting the associations between alleles and traits was set at *P* < 0.01. Finally, corrections for multiple comparisons were performed using the false discovery rate (FDR) with *Q* value in R [91]. The ratio of dominant (d) to additive (a) was used to measure the gene effect of the significant loci. Values of |d/a| ≤ 0.5 were defined as additive effects, 0.50 < |d/a| < 1.25 as partial or complete dominance, and |d/a| > 1.25 as under- or over dominance [92, 93]. The specific algorithm and formulas for calculating gene effect were as previously described. To identify the putative function of the unigene sequences containing the polymorphic microsatellite loci, the sequences were analyzed from NCBI (http:// www.ncbi.nlm.nih.gov) nonredundant protein database using BLASTX search.

## Supplementary information

**Additional file 1: Figure S1.** Principal component analysis of 192 individuals based on 30 EST-SSR markers.

**Additional file 2: Figure S2.** Q cluster analysis f 192 *S. oblata* basing on 9 Phenotypic traits.

**Additional file 3: Table S1.** Nine hundred thirty-two pairs of SSR marker information developed in the transcriptome of *S. oblata*.

**Additional file 4: Table S2.** Descriptive statistics for phenotypic traits measured in the trail of *S. oblata trees*.

**Additional file 5: Table S3.** Estimates of phenotypic correlations for nine traits in the association population.

**Additional file 6: Table S4.** The putative function of 17polymorphic EST-SSRs and their homologies to protein-coding genes.

**Additional file 7: Table S5.** Nine investigation traits of association population in this study and measurement standard.

## Data Availability

The primers designed in this article are included within the article and its additional files. The RNA-seq data that support the findings of this study have been deposited to the NCBI Sequence Read Archive (SRP063913, https://trace.ncbi.nlm.nih.gov/Traces/sra/?study=SRP063913). All the materials that support these findings do not contain wild resources, and all of them are cultivated germplasm resources of *S. oblata*. Beijing anjiming Labor Co., Ltd. is in full compliance with institutional, national or international guidelines and has obtained appropriate permissions and business licenses.

## Abbreviations

MAS: Marker-assisted selection
SSR: Simple sequence repeats
RAPD: Random amplified polymorphic DNA
AFLP: Amplified fragment length polymorphism
EST: Expressed sequence tag
NGS: Next-generation sequencing
*N*_*A*_: Observed number
*N*_*E*_: Effective number
*H*_*O*_: Observed heterozygosities
*H*_*E*_: Expected heterozygosities
I: Shannon’s information index
PIC: Polymorphism information content
MLM: Mixed linear model
FDR: False discovery rate
MCMC: Markov chain-Monte Carlo
LD: Linkage disequilibrium
MAF: Minor allele frequencies
HWE: Hardy-Weinberg equilibrium

## References

[1] Cui HX, Jiang GM, Zang SY (2004). The distribution, origin and evolution of *Syringa*. Bull Bot Res.

[2] Zhang SY, Cui HX (2000). Lilac. 1st ed.

[3] Wei ZP, Li QF. Study on the preservation, breeding and extension of Lilac germplasm resource. Part Acultural Sci. 2007;24(2):77–80.

[4] Meng X (2011). Collection and landscape application of *Syringa* germplasm resources in Beijing. Beijing Garden.

[5] Xue C, Jiao H, Zhang W, Sun Y, Qu S, Zhuang Z, He M (2015). Research progress of *Syringa* breeding. Tianjin Agric Sci.

[6] Dai RQ, Zhang L, Hu DQ, Zhao WG, Pan G, Liu L (2009). Research advance in plant molecular breeding. J Anhui Agric Sci.

[7] Du QZ, Xu BH, Pan W, Gong CR, Wang QS, Tian JX, Li BL, Zhang DQ (2013). Allelic variation in a cellulose synthase gene (*PtoCesA4*) associated with growth and wood properties in *Populus tomentosa*. G3 Genes Genom Genet.

[8] Fifippi CV, Aguirre N, Rivas JG, Zubrzycki J, Puebla A, Cordes D, Moreno MV, Fusari CM, Alvarez D, Heinz RA, Hopp HE, Paniego NB, Lia VV (2015). Population structure and genetic diversity characterization of a sunflower association mapping population using SSR and SNP markers. BMC Plant Biol.

[9] Ma KF, Sun LD,Cheng TR, Pan HT, Wang J, Zhang QX. Epigenetic variance, performing cooperative structure with genetics, Is Associated with Leaf Shape Traits in Widely Distributed Populations of Ornamental Tree *Prunus mume* Front Plant Sci 2018;9:41. 10.3389/fpls.2018.00041 PMC5797549 29441078

[10] Zheng TC, Qin B, Li SZ, Cai M, Pan HT, Wang J, Cheng TR, Zhang QX (2019). Screening of applicable SSR molecular markers linked to creeping trait in crape Myrtle. Forests..

[11] Wu J, Cheng FY, Cai CF, Zhong Y, Jie X. Association mapping for floral traits in cultivated *Paeonia rockii* based on SSR markers. Mol Gen Genomics. 2016;292(1):1–14. 10.1007/s00438-016-1266-0 27807670

[12] Su JS, Zhang F, Li PR, Guan ZY, Fang WM, Chen FD. Genetic variation and association mapping of waterlogging tolerance in chrysanthemum. Planta. 2016;244:1241–52. 10.1007/s00425-016-2583-6 27522648

[13] Yang Y, Xuan L, Yu CG, Wang ZY, Xu JH, Fan WC, Guo JB, Yin YL (2018). High-density genetic map construction and quantitative trait loci identification for growth traits in (*Taxodium distichum* var. *distichum* × *T. mucronatum*) × *T. mucronatum*. BMC Plant Biol.

[14] Zhang F, Kang JM, Long RC, Yu LX, Wang Z, Zhao ZX, Zhang TJ, Yang QC (2019). High-density linkage map construction and mapping QTL for yield and yield components in autotetraploid alfalfa using RAD-seq. BMC Plant Biol.

[15] Ming J, Gu WC (2006). Genetic diversity in natural populations of *Syringa oblata* detected by AFLP markers. Acta Horticulturae Sinica.

[16] Liao H, Gu W, Ming J (2009). Determining genetic diversity of natural population of *Syringa oblata* using allozyme markers. J Beijing Forest Univ.

[17] Jose AG, Concepción M, Francisco M. Trends in plant research using molecular markers. Planta. 2017;247:543–57. 10.1007/s00425-017-2829-y 29243155

[18] Gupta PK, Balyan HS, Sharma PC, Ramesh B (1996). Microsatellites in plants: a new class of molecular markers. Curr Sci.

[19] Kohn CV, Conrad K, Kramer M, Pooler M (2018). Genetic diversity of *Magnolia ashei* characterized by SSR markers. Conserv Genet.

[20] Liu Y, He D, Cai M, Tang W, Li XY, Pan HT, Zhang QX (2013). Development of microsatellite markers for *Lagerstroemia indica* (Lythraceae) and related species. Appl Plant Sci.

[21] Guo LL, Guo DL, Zhao W, Hou XG (2018). Newly developed SSR markers reveal genetic diversity and geographical clustering in *Paeonia suffruticosYea* based on flower colour. J Hortic Sci Biotechnol.

[22] Qi WC, Chen X, Fang PH, Shi SC, Li JJ, Liu XT, Gao XQ, Zhao N, Hao HY, Li YJ, Han YJ, Zhang Z (2018). Genomic and transcriptomic sequencing of *Rosa hybrida* provides microsatellite markers for breeding, flower trait improvement and taxonomy studies. BMC Plant Biol.

[23] Gadissa F, Tesfaye K, Dagne K, Geleta M (2018). Genetic diversity and population structure analyses of *Plectranthus edulis* (Vatke) Agnew collections from diverse agroecologies in Ethiopia using newly developed EST-SSRs marker system. BMC Genet.

[24] Pinto MV, Poornima HS, Sivaprasad V, Naik VG. A new set of mulberry-specific SSR markers for application in cultivar identification and DUS testing. J Genet. 2018;97. 29700272

[25] Wiersma PA, Deniz E, Shawkat A (2018). DNA fingerprinting of closely related cultivars of sweet cherry. J Am Soc Hortic Sci.

[26] Fenton ME, Owens BF, Lipka AE, Ortiz D, Tiede T, Mateos-Hernandez M, Ferruzzi MG, Rocheford T. High-density linkage mapping of vitamin E content in maize grain. Mol Breed. 2018;31.

[27] Li PR, Su JS, Guan ZY, Fang WM, Chen FD, Zhang F (2018). Association analysis of drought tolerance in cut chrysanthemum (*Chrysanthemum morifolium* Ramat.) at seedling stage. Biotech..

[28] Juntheikki-Palovaara I, Antonius K, Linde’n L, Korpelainen H (2013). Microsatellite markers for common lilac (*Syringa vulgaris* L.). Plant Genetic Resour.

[29] Lendvay B, Pedryc A, Höhn M (2013). Characterization of nuclear microsatellite markers for the narrow endemic *Syringa josikaea* Jacq. Fil. Ex Rchb. Notulae Botanicae Horti Agrobotanici.

[30] Park JH, Ahn SG, Choi YM, Oh HJ, Ahn DC, Kim JG, Kang JS, Choi YW, Jeong BR. Rose (*Rosa hybrida* L.) EST-derived microsatellite markers and their transferability to strawberry (*Fragaria* spp.). Sci Hortic. 2010;125:733–739.

[31] Ye YJ, Feng L, Liang XH, Liu TT, Cai M, Cheng TR, Wang J, Zhang QX, Pan HT (2019). Characterization, validation, and cross-species transferability of newly developed EST-SSR markers and their application for genetic evaluation in crape myrtle (*Lagerstroemia* spp). Mol Breed.

[32] Wang XL, Chen WC, Luo J, Yao ZX, Yu Q, Wang YL, Zhang SZ, Liu ZG, Zhang MR, Shen YM. Development of EST-SSR markers and their application in an analysis of the genetic diversity of the endangered species *Magnolia sinostellata*. Mol Gen Genomics. 2018;294:135–47. 10.1007/s00438-018-1493-7 30255205

[33] Zheng J, Hu ZH, Guan XL, Dou DQ, Bai G, Wang Y, Guo YT, Li W, Leng PS. Transcriptome analysis of *Syringa oblata* Lindl. inflorescence identifies genes associated with pigment biosynthesis and scent metabolism. PLoS One. 2015;10(11):e0142542. 10.1371/journal.pone.0142542 PMC4654506 26587670

[34] Temnykh S, DeClerck G, Lukashova A, Lipovich L, Cartinhour S, McCouch S (2001). Computational and experimental analysis of microsatellites in rice (*Oryza sativa* L.): frequency, length variation, transposon associations, and genetic marker potential. Genome Res.

[35] Evanno G, Regnaut S, Goudet J (2005). Detecting the number of clusters of individuals using the software STRUCTURE: a simulation study. Mol Ecol.

[36] Eckert AJ, Bower AD, Wegrzyn JL, Pande B, Jermstad KD, Krutovsky KV, StClair JB, Neale DB (2009). Association genetics of coastal Douglas fir (*Pseudotsuga menziesii var. menziesii*, Pinaceae). I. Cold hardiness related traits. Genetics.

[37] Wegrzyn JL, Eckert AJ, Choi M, Lee JM, Stanton BJ, Sykes R, Davis MF, Tsai CJ, Neale DB (2010). 2010. Association genetics of traits controlling lignin and cellulose biosynthesis in black cottonwood (*Populus trichocarpa*, Salicaceae) secondary xylem. New Phytol.

[38] Chen H, Wang L, Wang S, Liu C, Blair MW, Cheng X. Transcriptome sequencing of mung bean (*Vigna radiate* L.) genes and the identification of EST-SSR markers. PLoS One. 2015;10. 10.1371/journal.pone.0120273 PMC4382333 25830701

[39] An M, Deng M, Zheng SS, Song YG (2016). De novo transcriptome assembly and development of SSR markers of oaks *Quercus austrocochinchinensis* and *Q. kerrii* (*Fagaceae*). Tree Genet Genomes.

[40] Xing W, Liao JY, Cai MY, Xia QF, Liu Y, Zeng W (2017). De novo assembly of transcriptome from *Rhododendron latoucheae* Franch using Illumina sequencing and development of new EST-SSR markers for genetic diversity analysis in Rhododendron. Tree Genet Genomes.

[41] Ma JQ, Yao MZ, Ma CL, Wang XC, Jin JQ, Wang XM, Chen L (2014). Construction of a SSR-based genetic map and identification of QTLs for catechins content in tea plant (*Camellia sinensis*). PLoS One.

[42] Cai K, Zhu LF, Zhang KK, Li L, Zhao ZY, Zeng W, Lin XC (2019). Development and characterization of EST-SSR markers from RNA-Seq data in *Phyllostachys violascens*. Front Plant Sci.

[43] Ali N, Li DL, Eltahawy MS, Abdulmajid D, Bux L, Liu EB, Dang XJ, Hong DL (2020). Mining of favorable alleles for seed reserve utilization efficiency in *Oryza sativa* by means of association mapping. BMC Genet.

[44] Kumpatla S, Mukhopadhyay S (2005). Mining and survey of simple sequence repeats in expressed sequence tags of dicotyledonous species. Genome..

[45] Zhang ZY, Xie WG, Zhao YQ, Zhang JC, Wang N, Ntakirutimana F, Yan JJ, Wang YR (2019). EST-SSR marker development based on RNA-sequencing of *E sibiricus* and its application for phylogenetic relationships analysis of seventeen *Elymus* species. BMC Plant Biol.

[46] Sathyanarayana N, Pittala RK, Tripathi PK, Chopra R, Singh HR, Belamkar V, Bhardwa PK, Doyle JJ, Egan AN (2017). Transcriptomic resources for the medicinal legume *Mucuna pruriens*: de novo transcriptome assembly, annotation, identification and validation of EST-SSR markers. BMC Genomics.

[47] Wang Z, Yan H, Fu X, Li X, Gao H (2013). Development of simple sequence repeat markers and diversity analysis in alfalfa (*Medicago sativa* L.). Mol Biol Rep.

[48] Pan L, Xia Q, Quan Z, Liu H, Ke W, Ding Y (2010). Development of novel EST-SSRs from sacred lotus (*Nelumbo nucifera* Gaertn) and their utilization for the genetic diversity analysis of *N. nucifera*. J Hered.

[49] Wu J, Cai CF, Cheng FY, Cui HL, Zhou H (2014). Characterisation and development of EST-SSR markers in tree peony using transcriptome sequences. Mol Breed.

[50] Zhang MY, Fan L, Liu QZ (2014). A novel set of EST derived SSR markers for pear and cross-species transferability in Rosaceae. Plant Mol Biol Report.

[51] Sharma RK, Bhardwaj P, Negi R, Mohapatra T, Ahuja PS (2009). Identification, characterization and utilization of unigene derived microsatellite markers in tea (*Camellia sinensis* L.). BMC Plant Biol.

[52] Shi J, Huang SM, Zhan JP, Yu JY, Wang XF, Hua W, Liu SY, Liu GH, Wang HZ (2014). Genome-wide microsatellite characterization and marker development in the sequenced *Brassica* crop species. DNA Res.

[53] Varshney RK, Thiel T, Stein N, Langridge P, Graner A (2002). In silico analysis on frequency and distribution of microsatellites in ESTs of some cereal species. Cell Mol Biol Lett.

[54] Katti MV, Ranjekar PK, Gupta VS (2001). Differential distribution of simple sequence repeats in eukaryotic genome sequences. Mol Biol Evol.

[55] Chen JF, Li RH, Xia YS, Bai GH, Guo PG, Wang ZL, Zhang H, Siddique KHM. Development of EST-SSR markers in flowering Chinese cabbage (*Brassica campestris* L. ssp. chinensis var. utilis Tsen et Lee) based on denovo transcriptomic assemblies. PLoS One. 2017;12(9):e0184736. 10.1371/journal.pone.0184736 PMC5597223 28902884

[56] Tang SX, Okashah RA, Cordonnier-Pratt MM, Pratt LH, Johnson VE, Taylor CA, Arnold ML, Knapp SJ (2009). EST and EST-SSR marker resources for *Iris*. BMC Plant Biol.

[57] Han ZZ, Ma XY, Wei M, Zhao T, Zhan RT, Chen WW (2018). SSR marker development and intraspecific genetic divergence exploration of *Chrysanthemum indicum* based on transcriptome analysis. BMC Genomics.

[58] Kumar S, Shah N, Garg V, Bhatia S. Large scale in-silico identifification and characterization of simple sequence repeats (SSRs) from de novo assembled transcriptome of *Catharanthus roseus* (L.) G. Don. Plant Cell Rep. 2014;33(6):905–18. 10.1007/s00299-014-1569-8 24482265

[59] Yan ZZ, Wu F, Luo K, Zhang YF, Yan Q, Zhang YF, Wang YR, Zhang JY. Cross-species transferability of EST-SSR markers developed from the transcriptome of *Melilotus* and their application to population genetics research. Sci Rep. 2017;7(1):17959. 10.1038/s41598-017-18049-8 PMC5738344 29263338

[60] Yu HP, Cheng FY, Zhong Y, Cai CF, Wu J, Cui HL (2013). Development of simple sequence repeat (SSR) markers from *Paeonia ostii* to study the genetic relationships among tree peonies (Paeoniaceae). Sci Hortic.

[61] King RA, Harris SL, Karp A, Barker JH (2010). Characterization and inheritance of nuclear microsatellite loci for use in population studies of the allotetraploid Salix alba–Salix fragilis complex. Tree Genet Genomes.

[62] Ding J, Ali F, Chen G, Li H, Mahuku G, Yang N, Yan J (2015). Genome-wide association mapping reveals novel sources of resistance to northern corn leaf blight in maize. BMC Plant Biol.

[63] Wang X, Jia MH, Ghai P, Lee FN, Jia Y (2015). Genome-wide association of rice blast disease resistance and yield-related components of rice. Mol Plant-Microbe Interact.

[64] Pritchard JK, Stephens M, Donnelly P (2000). Inference of population structure using multilocus genotype data. Genetics.

[65] Du QZ, Pan W, Xu BH, Li BL, Zhang DQ (2013). Polymorphic simple sequence repeat (SSR) loci within cellulose synthase (*PtoCesA*) genes are associated with growth and wood properties in *Populus tomentosa*. New Phytol.

[66] Du QZ, Pan W, Tian JX, Li BL, Zhang DQ (2013). The UDP-Glucuronate decarboxylase gene family in *Populus*: structure, expression, and association genetics. PLoS One.

[67] Brown GR, Gill GP, Kuntz RJ, Langley CH, Neale DB (2004). Nucleotide diversity and linkage disequilibrium in loblolly pine. Proc Natl Acad Sci U S A.

[68] Dvornyk V, Sirvio A, Mikkonene M, Savolainen O (2002). Low nucleotide diversity at two phytochrome loci along a latitudinal cline in *Pinus sylvestris*. Mol Biol Evol.

[69] Neale DB, Savolainen O (2004). Association genetics of complex traits in conifers. Trends Plant Sci.

[70] Krutovsky KV, Neale DB (2005). Nucleotide diversity and linkage disequilibrium in cold-hardiness and wood quality-related candidate genes in Douglas-fir. Genetics.

[71] Gonzalez-Martinez SC, Wheeler NC, Ersoz E, Nelson CD, Neale DB (2007). Association genetics in *Pinus taeda* L. I Wood Prop Traits Genetics.

[72] Maryam F, Hossein SA, Ali K, Morteza A (2019). Phenotypic diversity among *Morus alba* var. *nigra* genotypes as revealed by multivariate analysis. Sci Hortic.

[73] Ming J, Gu WC (2006). Phenotypic variation of *Syringa oblata* Lindl. For Res.

[74] Zhang X, Wang YY, Che DD (2010). The Morphologyl characters and variation analysis of *Syringa oblata* in different biotope. Advances in ornamental horticulture of China.

[75] Zarbafi SS, Rabiei B, Ebadi AA, Ham JH (2020). Association mapping of traits related to leaf blast disease in rice (*Oryza sativa* L.). Australas Plant Pathol.

[76] Nie G, Tang L, Zhang YJ, Huang LK, Ma X, Cao X, Pan L, Zhang X, Zhang XQ (2017). Development of SSR markers based on transcriptome sequencing and association analysis with drought tolerance in perennial grass *Miscanthus* from China. Front Plant Sci.

[77] Yi Q, Liu YH, Hou XB, Zhang XG, Li H, Zhang JJ, Liu HM, Hu YF, Yu GW, Li YP, Wang YB, Huang YB (2019). Genetic dissection of yield-related traits and mid-parent heterosis for those traits in maize (*Zea mays* L.). BMC Plant Biol.

[78] Sun X, Du Z, Ren J, Amombo E, Hu T, Fu J (2015). Association of SSR markers with functional traits from heat stress in diverse tall fescue accessions. BMC Plant Biol.

[79] González-Martínez SC, Wheeler NC, Ersoz E, Nelson CD, Neale DB (2007). Association genetics in *Pinus taeda* LI wood property traits. Genetics.

[80] Beaulieu J, Doerksen T, Boyle B, Clément S, Deslauriers M, Beauseigle S, Rigault P (2011). Association genetics of wood physical traits in the conifer white spruce and relationships with gene expression. Genetics.

[81] Dillon SK, Brawner JT, Meder R, Lee DJ, Southerton SG (2012). Association genetics in *Corymbia citriodora* subsp variegata identifies single nucleotide polymorphisms affecting wood growth and cellulosic pulp yield. New Phytol.

[82] Porth I, Klapšte J, Skyba O, Hannemann J, McKown AD, Guy RD, Friedmann MC (2013). Genome-wide association mapping for wood characteristics in Populus identifies an array of candidate single nucleotide polymorphisms. New Phytol.

[83] Du QZ, Wang L, Zhou DL, Yang HJ, Gong CR, Pan W, Zhang DQ (2014). Allelic variation within the S-adenosyl-Lhomocysteine hydrolase gene family is associated with wood properties in Chinese white poplar (*Populus tomentosa*). BMC Genet.

[84] Slatkin M, Muirhead CA (1999). Overdominant alleles in a population of variable size. Genetics.

[85] Luo GY, Ye LF, Chen XB (2013). Research progress of *Arabidopsis* B3 transcription factor gene superfamily. Chem Life.

[86] Huang JP, Li HL, Chang Y (2012). Genome-wide analysis of ovate family proteins in *Arabidopsis*. J Northeast Agric Univ (English edition).

[87] Yeh FC, Yang RC, Boyle T, Ye ZH, Mao JX. POPGENE, version 132: the user friendly software for population genetic analysis. Molecular Biology and Biotechnology Centre. Canada: University of Alberta, Edmonton; 1999.

[88] Liu K, Muse SV (2005). PowerMarker: an integrated analysis environment for genetic marker analysis. Bioinformatics..

[89] Yu J, Pressoir G, Briggs WH, Bi IV, Yamasaki M, Doebley JF, Kresovich S (2006). A unified mixed-model method for association mapping that accounts for multiple levels of relatedness. Nat Genet.

[90] Hardy OJ, Vekemans X (2002). SPAGeDi: a versatile computer program to analyse spatial genetic structure at the individual or population levels. Mol Ecol Notes.

[91] Storey JD, Tibshirani R (2003). Statistical significance for genomewide studies. Proc Natl Acad Sci U S A.

[92] Eckert AJ, Bower AD, Wegrzyn JL, Pande B, Jermstad KD, Krutovsky KV, Neale DB (2009). Association genetics of coastal douglasfir (*Pseudotsuga menziesii var. menziesii*, Pinaceae) I. cold-hardiness related traits. Genetics.

[93] Wegrzyn JL, Eckert AJ, Choi M, Lee JM, Stanton BJ, Sykes R, Neale DB (2010). Association genetics of traits controlling lignin and cellulose biosynthesis in black cottonwood (*Populus trichocarpa*, Salicaceae) secondary xylem. New Phytol.

