# Supplementary figures and images for: Development of EST-SSR markers and association mapping with floral traits in Syringa oblata
Source: BMC Plant Biol. 2020 Sep 21;20:436. doi: 10.1186/s12870-020-02652-5 (PMC7507607; doi:10.1186/s12870-020-02652-5)

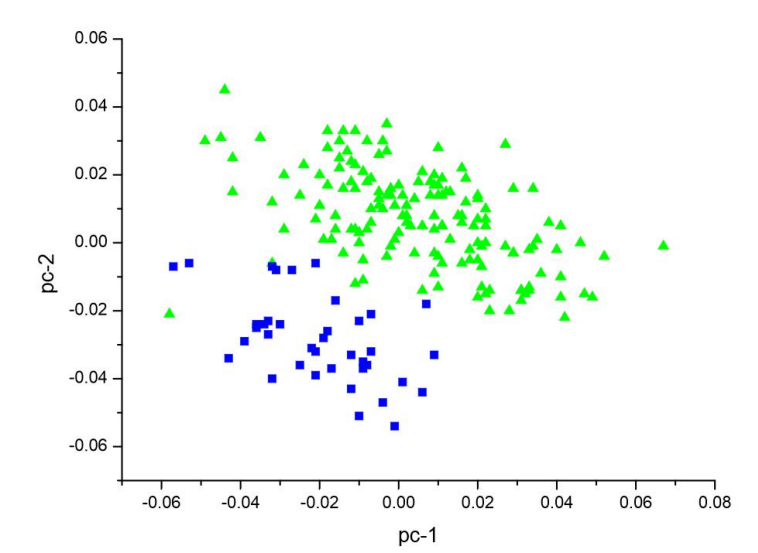


**Figure S1** Principal component analysis of 192 individuals based on 30 EST-SSR markers

Supplement: Supplementary file 1 — Additional file 1: Figure S1. Principal component analysis of 192 individuals based on 30 EST-SSR markers. [file 12870_2020_2652_MOESM1_ESM.doc]

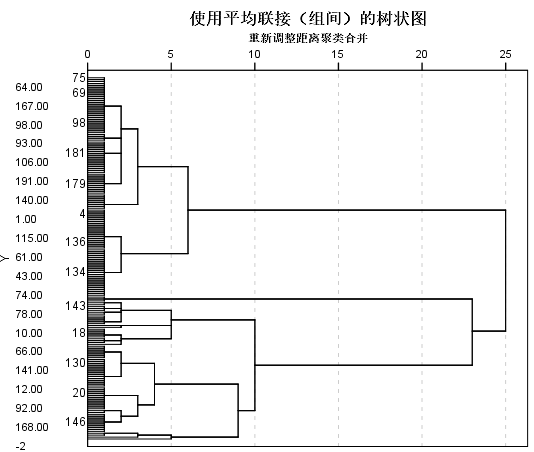


**Figure S2** Q cluster analysis f 192 *S. oblata* basing on 9 Phenotypic traits

Supplement: Supplementary file 2 — Additional file 2: Figure S2. Q cluster analysis f 192 S. oblata basing on 9 Phenotypic traits. [file 12870_2020_2652_MOESM2_ESM.doc]
