# Supplementary material for: Development of EST-SSR markers and association mapping with floral traits in Syringa oblata
Source: BMC Plant Biol. 2020 Sep 21;20:436. doi: 10.1186/s12870-020-02652-5 (PMC7507607; doi:10.1186/s12870-020-02652-5)
Supplement: Supplementary file 4 — Additional file 4: Table S2. Descriptive statistics for phenotypic traits measured in the trail of S. oblata trees. [file 12870_2020_2652_MOESM4_ESM.doc]

**Table S2 Descriptive statistics for phenotypic traits measured in the trail of *S. oblata* trees. IL, Inflorescence Length; IW, Inflorescence Width; CLL, Corolla Lobe Length; CLW, Corolla Lobe Width; WTL, Corolla Tube Length.**

| **Trait** | **Minimum** | **Maximum** | **Mean** | **Standard deviation** | **Variable coefficient** | ***F*-value** | ***P*-value** |
| --- | --- | --- | --- | --- | --- | --- | --- |
| **IL(mm)** | 44.50 | 180.50 | 89.95 | 27.19 | 30.22 | 29.76 | 0.00 |
| **IW(mm)** | 35.89 | 136.23 | 57.43 | 15.43 | 26.87 | 12.08 | 0.00 |
| **CLL(mm)** | 3.04 | 14.18 | 6.65 | 1.56 | 23.43 | 118.62 | 0.00 |
| **CLW(mm)** | 1.39 | 8.84 | 4.07 | 1.00 | 24.59 | 82.54 | 0.00 |
| **WTL(mm)** | 5.71 | 20.37 | 12.03 | 2.37 | 19.72 | 148.92 | 0.00 |
