# Supplementary material for: Development of EST-SSR markers and association mapping with floral traits in Syringa oblata
Source: BMC Plant Biol. 2020 Sep 21;20:436. doi: 10.1186/s12870-020-02652-5 (PMC7507607; doi:10.1186/s12870-020-02652-5)
Supplement: Supplementary file 5 — Additional file 5: Table S3. Estimates of phenotypic correlations for nine traits in the association population. [file 12870_2020_2652_MOESM5_ESM.doc]

**Table S3 Estimates of phenotypic correlations for nine traits in the association population. IL, Inflorescence Length; IW, Inflorescence Width; CLL, Corolla Lobe Length; CLW, Corolla Lobe Width; WTL, Corolla Tube Length; CLS, Corolla Lobe State; CLP, Corolla Lobe Periphery; F, Florescence; PC, Petal Color. *, *P* < 0.05; **, *P* < 0.01.**

| **Trait** | **IL** | **IW** | **CLL** | **CLW** | **WTL** | **CLS** | **CLP** | **F** | **PC** |
| --- | --- | --- | --- | --- | --- | --- | --- | --- | --- |
| **IL** | 1.000 |  |  |  |  |  |  |  |  |
| **IW** | 0.767** | 1.000 |  |  |  |  |  |  |  |
| **CLL** | 0.457** | 0.479** | 1.000 |  |  |  |  |  |  |
| **CLW** | 0.447** | 0.461** | 0.757** | 1.000 |  |  |  |  |  |
| **WTL** | 0.524** | 0.614** | 0.599** | 0.498** | 1.000 |  |  |  |  |
| **CLS** | -0.020 | -0.070 | -0.074 | -0.026 | -0.052 | 1.000 |  |  |  |
| **CLP** | 0.030 | 0.110 | 0.038 | 0.151* | 0.086 | 0.475** | 1.000 |  |  |
| **F** | 0.110 | 0.050 | -0.080 | 0.041 | 0.007 | 0.069 | 0.020 | 1.000 |  |
| **PC** | 0.040 | 0.060 | -0.086 | 0.015 | 0.024 | 0.159* | 0.177* | 0.070 | 1.000 |
