# Supplementary material for: Development of EST-SSR markers and association mapping with floral traits in Syringa oblata
Source: BMC Plant Biol. 2020 Sep 21;20:436. doi: 10.1186/s12870-020-02652-5 (PMC7507607; doi:10.1186/s12870-020-02652-5)
Supplement: Supplementary file 6 — Additional file 6: Table S4. The putative function of 17polymorphic EST-SSRs and their homologies to protein-coding genes. [file 12870_2020_2652_MOESM6_ESM.doc]

| **Primer name** | **Putative fuction** | ***E*-value** | **Accession number** |
| --- | --- | --- | --- |
| SO060 | hypothetical protein DVH24_016819 [*Malus domestica*] | 3E-04 | RXH73997.1 |
| SO104 | phosphatidylinositol/phosphatidylcholine transfer protein SFH6-like isoform X1 [*Olea europaea* var. *sylvestris*] | 3E-08 | XP_022896015.1 |
| SO112 | transketolase, chloroplastic [*Olea europaea* var. *sylvestris*] | 0.0 | XP_022886335.1 |
| SO208 | pentatricopeptide repeat-containing protein At1g26460, mitochondrial [*Olea europaea* var. *sylvestris*] | 0.0 | XP_022891171.1 |
| SO311 | uncharacterized protein LOC111405302 [*Olea europaea* var. *sylvestris*] | 0.0 | XP_022889890.1 |
| SO331 | uncharacterized protein LOC111403452 [*Olea europaea* var. *sylvestris*] | 0.001 | XP_022887734.1 |
| SO415 | transcription repressor OFP15-like [*Olea europaea* var. *sylvestris*] | 3E-64 | XP_022842089.1 |
| SO503 | none |  |  |
| SO505 | none |  |  |
| SO531 | protein MLN51 homolog [*Olea europaea* var. *sylvestris*] | 0.0 | XP_022889688.1 |
| SO608 | Copia protein [*Cajanus cajan*] | 5E-37 | KYP62853.1 |
| SO627 | hypothetical protein CUMW_142460 [*Citrus unshiu*] | 1.1 | GAY52521.1 |
| SO649 | E3 ubiquitin-protein ligase At1g63170-like [*Olea europaea* var. *sylvestris*] | 4E-71 | XP_022864126.1 |
| SO663 | B3 domain-containing protein Os03g0120900-like [*Olea europaea* var. *sylvestris*] | 4E-165 | XP_022892987.1 |
| SO695 | none |  |  |
| SO790 | none |  |  |
| SO805 | uncharacterized protein LOC111409858 [*Olea europaea* var. *sylvestris*] | 6E-87 | XP_022895709.1 |

**Table S4** The putative function of 17polymorphic EST-SSRs and their homologies to protein-coding genes.
