# Supplementary material for: Development of EST-SSR markers and association mapping with floral traits in Syringa oblata
Source: BMC Plant Biol. 2020 Sep 21;20:436. doi: 10.1186/s12870-020-02652-5 (PMC7507607; doi:10.1186/s12870-020-02652-5)
Supplement: Supplementary file 7 — Additional file 7: Table S5. Nine investigation traits of association population in this study and measurement standard. [file 12870_2020_2652_MOESM7_ESM.doc]

Table S5 9 investigation traits of association population in this study and measurement standard.

| **Trait** | **Measurement standard** | **Data type** |
| --- | --- | --- |
| Inflorescence Length (IL) | Length of whole inflorescence from top to bottom（mm) | Quantitative |
| Inflorescence Width (IW) | Width between ends of the widest part of the whole inflorescence（mm) | Quantitative |
| Corolla Lobe Length (CLL) | Length of corolla lobe of single flower（mm) | Quantitative |
| Corolla Lobe Width (CLW) | Width of corolla lobe of single flower（mm) | Quantitative |
| Corolla Tube Length (WTL) | Length of corolla tube of single flower（mm) | Quantitative |
| Corolla Lobe State (CLS) | Horizontal; zigzag; oblique expansion | Classification |
| Corolla Lobe Periphery (CLP) | Flatness; undulating; spoon type | Classification |
| Petal Color (PC) | White; pale pink; purple; purplish red; purplish pink; violet | Classification |
| Florescence (F) | Early; middle; late | Classification |
